# Development of a preoperative prediction model for vascular recanalization in cervical artery dissection based on HR-VWI radiomics features combined with clinical risk factors

**DOI:** 10.3389/fphys.2026.1886681

**Published:** 2026-09-14

**Authors:** Tao Li, Yue Ma, Lei Wang, Meiling Zhang, Xueliang Tian, Xuanxiao Zhang, Wenjing Gu, Hongwei Zhou, Jiaxin Liu

**Affiliations:** 1 Department of Radiology, The First Hospital of Jilin University, Changchun, China; 2 Department of Radiology, Liaoyuan Central Hospital, Liaoyuan, China; 3 Department of Breast Surgery, The First Hospital of Jilin University, Changchun, China; 4 Department of Otorlaryngology, The First Hospital of Jilin University, Changchun, China

**Keywords:** artificial intelligence, CEAD, cervical artery dissection, high-resolution vessel wall imaging, multimodal imaging, prognostic prediction, radiomics, risk stratification

## Abstract

**Objective:**

To explore the feasibility of constructing a prediction model for vascular recanalization in cervical artery dissection (CeAD) based on high-resolution vessel wall imaging (HR-VWI) radiomics features combined with clinical risk factors.

**Methods:**

A total of 110 patients with CeAD were retrospectively enrolled. Clinical baseline data and HR-VWI images were collected. The Radiomics Intelligent Analysis Software (RIAS) was used for image preprocessing, ROI segmentation, and radiomics feature extraction and screening. Combined with clinical factors, multiple prediction models including random forest, gradient boosting decision tree, and logistic regression (LR) were constructed. Model performance was evaluated using AUC, calibration, Brier score, and decision curve analysis (DCA).

**Results:**

A total of 11 features (6 radiomics + 5 clinical) were selected through a multi-step workflow (ICC filtering → t-test → LASSO → backward AIC selection). The LR model achieved the best performance, with an AUC of 0.757 (95% CI: 0.531–0.983) and average precision of 0.95 in the independent test set. The model showed acceptable calibration (Brier score = 0.129; intercept = 0.913; slope = 0.896) and favorable clinical utility by DCA. Hyperuricemia, hyperlipidemia, smoking, headache, stenosis degree, and specific radiomics features were independent predictors of recanalization. Wavelet-HLH_glszm_Small Area High Gray Level Emphasis and wavelet-LHH_glcm_SumEntropy showed the most significant predictive effects. However, given the limited sample size and few events, these results should be interpreted as preliminary.

**Conclusion:**

The combined LR model shows moderate discriminative ability for predicting vascular recanalization in CeAD. As a proof-of-concept study, it provides preliminary reference for prognostic assessment, while external validation in larger multi-center cohorts is warranted before clinical implementation.

## 1 Introduction

Cervical artery dissection (CeAD) is a cerebrovascular disease of significant clinical importance, characterized by carotid intimal tear or arterial wall rupture leading to intramural hematoma formation, which can further cause serious complications such as intraluminal thrombosis, vascular stenosis, occlusion or pseudoaneurysm (Yaghi et al., 2024). CeAD accounts for approximately 2% of all ischemic strokes, but this proportion increases significantly to 15%-25% in young stroke patients under 50 years of age (Putaala et al., 2009; Béjot et al., 2014). Epidemiological studies have shown that the overall incidence of CeAD is approximately 4.69 cases per 100,000 person-years, with the incidence of internal cervical artery dissection and vertebral artery dissection being 2.43 and 2.01 cases per 100,000 person-years, respectively. The disease is characterized by high mortality, high disability rate and high recurrence rate, which seriously endangers the health of residents in China (Lee et al., 2006; Debette and Leys, 2009; Putaala et al., 2009).Current studies indicate that most CeAD cases are spontaneous, but approximately 90% of traumatic dissections are caused by minor trauma, including daily activities such as neck massage, weightlifting, yoga and childbirth (Keser et al., 2022). In addition, multiple studies have identified various potential risk factors, such as recent infection, pregnancy status, oral contraceptive use, smoking history, migraine, elongated styloid process, vascular anatomical variations and genetic susceptibility. However, the relationship between these factors and vascular recanalization after CeAD remains to be further confirmed. Clinically, due to the relative rarity of CeAD, existing evidence mainly comes from case reports and case series studies, and the lack of support from large-scale randomized controlled trials has led to the absence of unified international diagnosis and treatment consensus (Keser et al., 2022).Currently, according to the recommendations of domestic and foreign guidelines, thrombolytic therapy for CeAD lumens, antiplatelet and anticoagulant therapy, and endovascular intervention are still the recommended treatment modalities for CeAD patients. However, clinical observations have found that some patients do not respond ideally to existing treatment regimens, and a large number of patients still cannot benefit from their application (Keser et al., 2022; Yaghi et al., 2024). As a key indicator for evaluating treatment efficacy, recanalization rate is affected by multiple factors, but systematic studies on the influencing factors of recanalization after CeAD are still limited. Therefore, in-depth study of the influencing factors of CeAD recanalization and guiding clinical decision-making through personalized vascular recanalization prediction is an important way to improve the prognosis of CeAD patients.Traditional prognostic assessment of CeAD mainly relies on clinicians’ subjective judgment of patients’ symptoms, signs and conventional imaging manifestations, which has limitations such as large inter-observer variability and limited predictive efficacy. Ultrasound, as the most commonly used follow-up method, can dynamically observe changes in vascular lumen, but cannot clearly display vascular wall structure and intramural hematoma characteristics, making it difficult to comprehensively evaluate the pathological state of dissection. Computed tomography angiography (CTA) can provide good vascular anatomical information, but it has ionizing radiation and limited resolution of soft tissue in the vessel wall (Baradaran and Gupta, 2020). In contrast, magnetic resonance high-resolution vessel wall imaging (HR-VWI) can clearly display the fine structure of the vessel wall, the morphology, signal characteristics and enhancement pattern of intramural hematoma, and has become the gold standard for the diagnosis and evaluation of CeAD (Habs et al., 2011; Hunter et al., 2012; Luo et al., 2016; Hashimoto et al., 2021; Ou et al., 2021). However, current prognostic studies of CeAD based on HR-VWI mostly focus on the analysis of qualitative imaging features, such as intramural hematoma signal intensity, intimal flap and double lumen sign, and have not fully explored the quantitative biological information contained in the images. Radiomics technology, as an emerging medical image analysis method, can extract hundreds to thousands of quantitative features from medical images with high throughput and mine deep microscopic structural information that cannot be recognized by the naked eye. It has shown great potential in the diagnosis, efficacy evaluation and prognosis prediction of tumors, cardiovascular and nervous system diseases (Lambin et al., 2012). However, radiomics studies also face challenges such as standardization of image acquisition, reproducibility of feature extraction, interpretability of models, and generalizability across centers and scanners. Standardized radiomics workflows and rigorous validation are essential for translating radiomics models into clinical practice (Lambin et al., 2017).

## 2 Materials and methods

### 2.1 Study subjects

This study adopted a retrospective research design and enrolled patients with clinically confirmed CeAD at the First Hospital of Jilin University from January 1, 2014 to December 31, 2024. All case screening processes and data collection work strictly followed the pre-formulated research protocol, which was approved by the Ethics Committee of the First Hospital of Jilin University (Approval No.: 2024-MS-142). Considering the inherent characteristics of this retrospective study, the Ethics Committee agreed to waive the signing of informed consent forms for subjects, and the entire research process strictly adhered to the relevant ethical norms in the *Declaration of Helsinki*.

#### 2.1.1 Inclusion criteria

(1) Clinically highly suspected CeAD and completed high-resolution vessel wall imaging within 48 hours of admission; (2) Clearly diagnosed with CeAD by high-resolution magnetic resonance vessel wall imaging, with lesions involving the internal carotid artery or vertebral artery and having typical imaging features; (3) Having complete clinical baseline data and clear outcome event records during the follow-up period.

#### 2.1.2 Exclusion criteria

(1) Imaging data with artifacts, missing phenomena, or data quality not meeting the basic requirements for radiomics feature extraction and analysis; (2) Complicated with other severe diseases or complications that may affect outcome judgment, such as intracranial tumors, vascular malformations, severe infections, etc.; (3) Having a history of previous related treatments such as neck surgery, interventional therapy or radiotherapy. (4) Patients who underwent endovascular stenting or interventional treatment during follow-up, as the vascular status was modified by intervention and could not reflect spontaneous recanalization.

### 2.2 Data collection

#### 2.2.1 Clinical data

Two trained neurologists independently reviewed the electronic medical record system to extract patients’ clinical baseline data, including: (1) Demographic information: age, gender; (2) Past medical history: hypertension, diabetes, coronary heart disease, stroke, migraine, hyperlipidemia, hyperuricemia, hyperhomocysteinemia; (3) Lifestyle: smoking history (defined as cumulative smoking ≥ 100 cigarettes), drinking history (defined as drinking ≥ 3 times per week for ≥ 1 year); (4) Family history: family history of cerebrovascular disease in first-degree relatives; (5) Clinical characteristics: infection history (within 2 weeks before onset), trauma history (within 1 week before onset), initial symptoms (headache, dizziness, limb weakness, speech disorder, etc.), time from onset to admission; (6) Treatment modalities: antiplatelet therapy, anticoagulant therapy, endovascular intervention. For cases with discrepancies during data extraction, consensus was reached after discussion (**Figure 10**).

#### 2.2.2 Imaging data

All patients underwent HR-VWI examination using a 3.0T magnetic resonance scanner (Ingenia, Philips Healthcare, Best, Netherlands) with an 8-channel head and neck combined coil. The scanning sequence included three-dimensional T1-weighted imaging (3D-T1WI) with the following specific scanning parameters: TR = 350 ms, TE = 19.1 ms, flip angle = 90°, FOV = 220 mm × 220 mm, matrix = 320 × 320, slice thickness = 1.4 mm, slice gap = 0.7 mm, scanning time approximately 5 minutes. All images were exported in DICOM format for subsequent imaging evaluation and radiomics analysis. All images were acquired on a single scanner with a standardized protocol, which minimized inter-scanner variability. Given the single-center design, no cross-scanner harmonization (e.g., ComBat) was applied. The potential impact of this on model generalizability is addressed in the Discussion.

### 2.3 Imaging evaluation

#### 2.3.1 Signal evaluation of intramural hematoma

On the axial maximum slice where the intramural hematoma was most clearly displayed in the 3D-T1WI sequence, two experienced radiologists independently manually delineated the region of interest and measured the signal intensity of the hematoma area. The signal intensity of adjacent muscle tissue (such as sternocleidomastoid muscle) at the same slice was used as an internal reference to calculate the relative signal intensity, with the formula: Relative signal intensity = Average signal intensity of intramural hematoma/Average signal intensity of adjacent muscle.

According to the numerical range of relative signal intensity, the signal intensity of intramural hematoma was divided into three grades: relative signal intensity ≥ 3 was defined as extremely high signal; relative signal intensity between 2 and 3 was defined as slightly high signal; relative signal intensity between 1 and 2 was defined as iso-signal. For cases with discrepant evaluation results, consensus was reached through negotiation or adjudication by a third senior physician, As shown in **Figure 1**.

**Figure 1:**
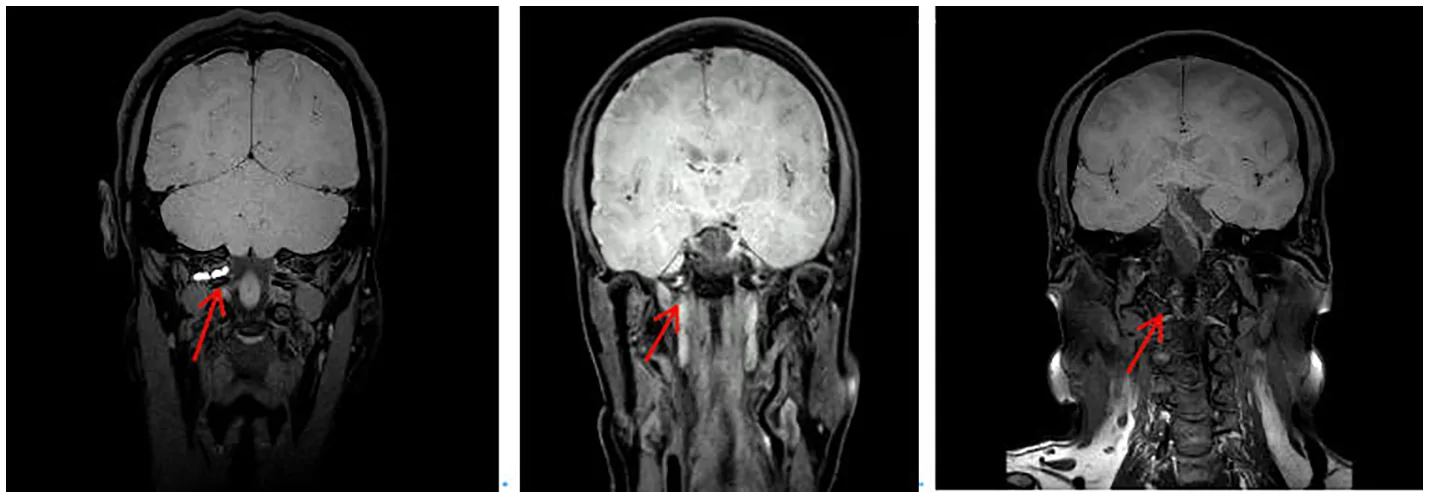
Representative coronal high-resolution vessel wall imaging (HR-VWI) of cervical artery dissection. Three consecutive coronal T1-weighted HR-VWI images demonstrate the full extent of cervical artery dissection from the skull base to the cervical segment. Red arrows point to the dissection lesions with intramural hematoma, which manifest as circumferential vessel wall thickening and hyperintense signal on T1-weighted sequence.

Inter-observer agreement for qualitative imaging assessments (signal grade, stenosis grade) was evaluated using Cohen’s kappa statistic, and agreement for continuous measurements (relative signal intensity, dissection length) was evaluated using the intraclass correlation coefficient (ICC). The kappa values for signal intensity grading and stenosis grading were 0.78 and 0.82, respectively, and the ICC values for relative signal intensity and dissection length were 0.85 and 0.83, respectively, all indicating good to excellent inter-observer reproducibility.

#### 2.3.2 Evaluation of luminal stenosis degree

Relevant measurements were performed on coronal maximum intensity projection reconstruction images of the 3D-T1WI sequence. According to the North American Symptomatic Carotid Endarterectomy Trial (NASCET) criteria, the vascular stenosis rate was calculated by the following formula:


Stenosis rate =[1−(minimum lumen diameter at the stenotic site/diameter of the distal normal lumen)]× 100%

According to the calculated stenosis rate values, the degree of luminal stenosis was divided into four grades: stenosis rate < 50% was defined as mild stenosis; stenosis rate between 50% and 69% was defined as moderate stenosis; stenosis rate between 70% and 99% was defined as severe stenosis; stenosis rate of 100% with disappearance of distal blood flow signal was defined as occlusion. All measurements were independently completed by two radiologists without knowledge of clinical outcomes. For cases with large differences in measurement results, consensus was reached through negotiation or review and adjudication by a third senior physician.

#### 2.3.3 Length of dissection-involved vessel

The high-resolution magnetic resonance imaging data of all patients were imported into the post-processing workstation, and the measurements were independently completed by two radiologists without knowledge of clinical outcomes. On the 3D-T1WI sequence, the morphology and extent of intramural hematoma were observed slice by slice. The slice where the hematoma was most clearly displayed and had the longest longitudinal extension was selected as the measurement plane, and the actual length of dissection involvement was measured along the course of the vessel, recorded in centimeters (cm) with two decimal places retained. The length of dissection was defined as the linear distance from the starting end to the terminating end of the hematoma along the vessel centerline. If the dissection was segmentally distributed, the length of each segment was measured separately and the sum was calculated. For cases with discrepant measurement results, consensus was reached through negotiation or review and adjudication by a third senior physician.

### 2.4 Radiomics analysis

This study was designed and reported in accordance with the Radiomics Quality Score (RQS) and TRIPOD guidelines. The radiomics workflow is illustrated in **Supplementary Figure 1**, and detailed procedures are described below.

#### 2.4.1 Region of interest segmentation and data preprocessing

The Radiomics Intelligent Analysis Software (RIAS, http://www.riascloud.com/main/indexlink), an in-house developed radiomics platform, was used for image preprocessing, ROI segmentation, and feature extraction (Van Griethuysen et al., 2017; Li et al., 2020).

Image preprocessing. All images were resampled to a uniform voxel size of 1.0 × 1.0 × 1.0 mm³ using B-spline interpolation to standardize spatial resolution. Signal intensity normalization was performed by mapping voxel values to the range [0, 255] based on the 1st and 99th percentiles of the image intensity histogram, thereby reducing the influence of extreme values and scanner-related intensity differences.

ROI delineation. The region of interest (ROI) was manually delineated on consecutive axial slices covering the entire intramural hematoma and thickened vessel wall on 3D-T1-weighted images. Of note, the ROI was strictly confined to the pathological vessel wall and intramural hematoma; adjacent normal reference tissue (e.g., sternocleidomastoid muscle) was NOT included within the ROI boundary. Three board-certified radiologists (with 5, 8, and 12 years of experience in neuroradiology, respectively) independently performed segmentation in a blinded manner. The final ROI was defined as the volume agreed upon by at least two radiologists; discrepancies were resolved by consensus or adjudication by a third senior radiologist.

Segmentation reproducibility. To assess inter-observer reproducibility of feature extraction, intraclass correlation coefficients (ICCs) were calculated based on segmentations from the three radiologists in a randomly selected subset of 30 patients. Only features with ICC > 0.75 (indicating good to excellent reproducibility) were retained for subsequent analysis. Intra-observer reproducibility was also evaluated by having one radiologist repeat the segmentation after a 4-week interval, and consistent results were observed (ICC > 0.80 for all retained features).

#### 2.4.2 Extraction, screening and preprocessing of radiomics features

Using the RIAS platform (http://www.riascloud.com/main/index), a total of 1,453 radiomics features were extracted from each VOI. These features encompassed seven categories: (1) 17 shape features describing the geometric properties of the lesion (volume, surface area, sphericity, maximum diameter, compactness, etc.); (2) 285 first-order statistical features characterizing the voxel intensity distribution (mean, median, kurtosis, skewness, percentiles, energy, entropy, etc.); (3) 360 gray level co-occurrence matrix (GLCM) features capturing second-order texture patterns; (4) 240 gray level size zone matrix (GLSZM) features; (5) 240 gray level run length matrix (GLRLM) features; (6) 210 gray level dependence matrix (GLDM) features; and (7) 75 neighborhood gray tone difference matrix (NGTDM) features. All first-order and texture features were computed on the original image as well as on multiple filter-transformed versions, including exponential, logarithm, square, square-root, and wavelet transforms (8 sub-bands: HLH, LHH, HHH, LLL, HLL, LLH, LHL, HHL).

Feature screening workflow. A multi-step feature screening strategy was applied, strictly nested within each cross-validation fold to avoid data leakage:

Step 1 – ICC filtering: Features with ICC ≤ 0.75 (inter-observer reproducibility) were excluded. After ICC filtering, 586 features with good to excellent reproducibility were retained.

Step 2 – Univariate t-test: Student’s t-test was used to identify features with significant between-group differences (recanalization vs. non-recanalization). Features with P < 0.05 were retained for further analysis.

Step 3 – LASSO regression: Least absolute shrinkage and selection operator (LASSO) logistic regression with 10-fold cross-validation was applied to further select the most predictive features and reduce multicollinearity. The optimal regularization parameter λ was determined based on the minimum mean squared error criterion.

Step 4 – Univariate logistic regression: Each remaining feature was entered into a univariate logistic regression model, and features with P < 0.2 were retained for multivariate modeling, following established methodological norms.

Step 5 – Backward stepwise selection with AIC: Backward stepwise selection based on the Akaike Information Criterion (AIC) was applied to optimize the final multivariate model. AIC balances model goodness-of-fit with parsimony, penalizing overly complex models to reduce overfitting risk.

Data normalization. Feature standardization (z-score normalization) was performed using the mean and standard deviation calculated from the training set only, and the same normalization parameters were applied to the test set to avoid data leakage.

### 2.5 Construction and evaluation of prediction models

Following the principle of randomization, the total dataset was divided into two parts in a 7:3 ratio: 70% of the samples (77 cases in total) were set as the cross-validation set for model training, feature weight optimization and internal validation; the remaining 30% of the samples (33 cases in total) were set as the independent test set, which did not participate in the model training, feature screening and parameter tuning processes throughout, and was only used for unbiased evaluation of the generalization ability of the final model. For the cross-validation set, a 5-fold cross-validation (5-fold CV) strategy was adopted for iterative training and internal validation.

Based on the optimal radiomics features and clinical features obtained through the strict screening process described above, multiple paradigms of models were selected for comparative analysis, specifically including: Logistic Regression (LR), Support Vector Machine (SVM), Random Forest Classifier (RFC), Gradient Boosting Decision Tree (GBDT), Multi-Layer Perceptron (MLP), and a stacked ensemble model (Stacker) constructed based on the above base models.

Hyperparameter tuning was performed for all non-linear models via grid search based on 5-fold cross-validation, with the validation set AUC as the optimization target. The search space and final optimal hyperparameters for each model are summarized in **Supplementary Table 1**.

Model calibration and clinical utility. In addition to discrimination performance, model calibration was assessed using: (1) calibration curves plotting observed event rates against predicted probabilities; (2) the calibration intercept (a measure of overall bias, with 0 indicating perfect calibration); (3) the calibration slope (a measure of spread, with 1 indicating perfect calibration); and (4) the Brier score (mean squared difference between predicted probabilities and observed outcomes, range 0–1, with lower values indicating better calibration). Clinical utility was evaluated using decision curve analysis (DCA), which quantifies the net benefit of the model across a range of threshold probabilities, compared with the “treat-all” and “treat-none” strategies.

### 2.6 Statistical analysis

SPSS 26.0 statistical software was used for data analysis. Categorical variables were statistically described as the number of cases and percentage (%), and inter-group comparisons were performed using the chi-square test or Fisher’s exact test according to the theoretical frequency. Age conformed to a normal distribution, was expressed as mean ± standard deviation, and the difference between the recanalization group and the non-recanalization group was compared using the independent samples t-test. Time from symptom onset to admission and length of dissection-involved vessel did not conform to a normal distribution, were expressed as median and interquartile range (P25, P75), and inter-group comparisons were performed using the Mann-Whitney U test.

To systematically quantify and evaluate the discrimination efficacy, internal stability and extrapolation generalization ability of the 6 constructed models, receiver operating characteristic (ROC) curves were plotted for the training set, validation set and independent test set divided in the model construction process, and the corresponding area under the curve (AUC) was calculated. Finally, the model with good diagnostic ability and clinical benefit was selected.

Univariate logistic regression was performed for all candidate variables, and a P-value threshold of 0.2 was set for preliminary screening following established methodological norms. Detailed univariate regression results are presented in **Supplementary Table 2**.

All performance metrics (AUC, sensitivity, specificity, precision, recall, F1-score, balanced accuracy, NPV, and PR-AUC) are reported with their 95% confidence intervals (CIs), calculated using the bootstrap method with 2,000 resamples. Calibration metrics (intercept, slope, Brier score) were also computed with bootstrap 95% CIs. All statistical analyses were performed using R software (version 4.2.3) with the rms, glmnet, pROC, and DecisionCurve packages, and SPSS 26.0. Model development and evaluation followed the established framework for clinical prediction models as previously described (Steyerberg and Vergouwe, 2014).

### 2.7 Outcome assessment

Vascular recanalization status during follow-up was defined as the primary outcome of this study.

- Definition of recanalization: Recanalization was defined as a composite endpoint including both complete recanalization and partial recanalization. Complete recanalization was defined as residual stenosis < 30% with basically normal lumen morphology. Partial recanalization was defined as a reduction in stenosis degree of ≥ 20% compared with baseline. Patients with no improvement, progression of stenosis or persistent occlusion were classified into the non-recanalization group.
- Follow-up imaging modality: Follow-up evaluation was mainly performed by color Doppler ultrasound, and supplemented by computed tomography angiography (CTA) or magnetic resonance angiography (MRA) when necessary. The final recanalization status was adjudicated based on the most comprehensive follow-up imaging.
- Follow-up schedule: The median follow-up duration was 6 months (interquartile range: 3–9 months), and the primary outcome was assessed at the 6-month follow-up. Given that most recanalization occurs within 6 months after onset and the binary outcome is clinically intuitive, binary logistic regression was adopted in this exploratory study. The limitation of non-uniform follow-up intervals is acknowledged, and time-to-event analysis will be applied in future studies with standardized follow-up protocols.
- Blinding of outcome assessment: Two independent radiologists who were blinded to clinical data and model results independently adjudicated the recanalization outcome. Discrepancies were resolved by consensus or by a third senior radiologist.
- Handling of interventional cases: Patients who received endovascular stenting or interventional treatment during follow-up were excluded from the final analysis, as described in the exclusion criteria.

## 3 Results

### 3.1 Demographic and clinical characteristics of subjects

A total of 110 patients were enrolled in this study, and were divided into the recanalization group (85 cases) and the non-recanalization group (25 cases) according to the vascular recanalization status during the follow-up period. Regarding lesion distribution, internal cervical artery dissection accounted for 68.2% (75/110) of cases, vertebral artery dissection accounted for 27.3% (30/110), and simultaneous multi-segment involvement accounted for 4.5% (5/110).The comparison of demographic and clinical characteristics between the two groups is shown in **Table 1**.

**Table 1:** Comparison of baseline characteristics between patients in the recanalization group and non-recanalization group.

Item	Recanalization group (n=85)	Non-recanalization group (n=25)	t/χ²/Z value	P value
Age (years, $\bar{\text{x}}$±s)	45.52 ± 13.10	48.84 ± 13.54	-1.107	0.271
Male [n (%)]	54 (63.53)	22 (88.0)	5.6	0.018*
Hypertension [n (%)]	29 (34.12)	11 (44.0)	0.80	0.372
Diabetes [n (%)]	6 (7.06)	3 (12.00)	1.07	0.300
History of stroke [n (%)]	42 (49.41)	16 (64.00)	1.65	0.198
Migraine [n (%)]	36 (42.35)	5 (20.0)	4.13	0.042*
Coronary heart disease [n (%)]	9 (10.59)	3 (12.00)	0.04	0.842
Hyperlipidemia [n (%)]	26 (30.59)	12 (48.00)	2.42	0.120
Smoking [n (%)]	32 (37.65)	17 (68.00)	7.19	0.007*
Drinking [n (%)]	20 (23.53)	11 (44.00)	3.45	0.063
Trauma history [n (%)]	7 (8.24)	0 (0.00)	0.71	0.400
Family history [n (%)]	20 (23.53)	2 (8.00)	0.05	0.829
Infection history [n (%)]	21 (27.06)	2 (8.00)	4.02	0.045*
Time from symptom onset to admission [days, M(P25, P75)]	10 (7, 12.67)	12 (8.9, 16)	-0.984	0.325
Intimal flap [n (%)]	19 (22.35)	5 (20.00)	0.06	0.804
Double lumen sign [n (%)]	40 (47.06)	9 (36.00)	0.51	0.476
Intraluminal thrombus [n (%)]	21 (24.71)	5 (20.00)	0.25	0.615
Dissection length [cm, M(P25, P75)]	3.2 (1.6, 4.7)	3 (1.25, 5.25)	-0.168	0.867
Signal type of intramural hematoma [n (%)]	–	–	4.146	0.126
Iso-signal	18 (21.2)	3 (12.0)	–	–
Slightly high signal	20 (23.5)	8 (32.0)	–	–
High signal	47 (55.3)	14 (56.0)	–	–
Degree of vascular stenosis [n (%)]	–	–	18.852	0.001*
Mild	22 (25.9)	1 (4.0)	–	–
Moderate	18 (21.2)	5 (20.0)	–	–
Severe	28 (32.9)	8 (32.0)	–	–
Occlusion	17 (20.0)	11 (44.0)	–	–
Antiplatelet therapy [n (%)]	77 (91.6)	22 (88)	0.30	0.584
Anticoagulant therapy [n (%)]	30 (35.29)	5 (20.00)	2.08	0.149

*: P < 0.05; -: No statistical value

This table compares the differences in demographic characteristics, clinical risk factors, medical history, imaging features, and treatment strategies between the recanalization group (n=85) and the non-recanalization group (n=25). Continuous variables are presented as mean ± standard deviation ( $\bar{x}\;\pm \;s$) for age and analyzed using the t-test; time from symptom onset to admission and dissection length are presented as median (interquartile range) [M(P25, P75)] and analyzed using the Mann-Whitney U test. Categorical variables are presented as counts (percentages) [n (%)] and analyzed using the chi-square test or Fisher’s exact test. * indicates P < 0.05, denoting a statistically significant difference; “−” indicates that no statistical value was available.

### 3.2 Data distribution

Using the random grouping method with a random seed of 104, the total dataset was divided into the cross-validation set and the independent test set in a 7.0:3.0 ratio. The cross-validation set included 77 cases, with 20 non-recanalization cases and 57 recanalization cases; the independent test set included 33 cases, with 5 non-recanalization cases and 28 recanalization cases. As shown in **Figure 2**.

**Figure 2:**
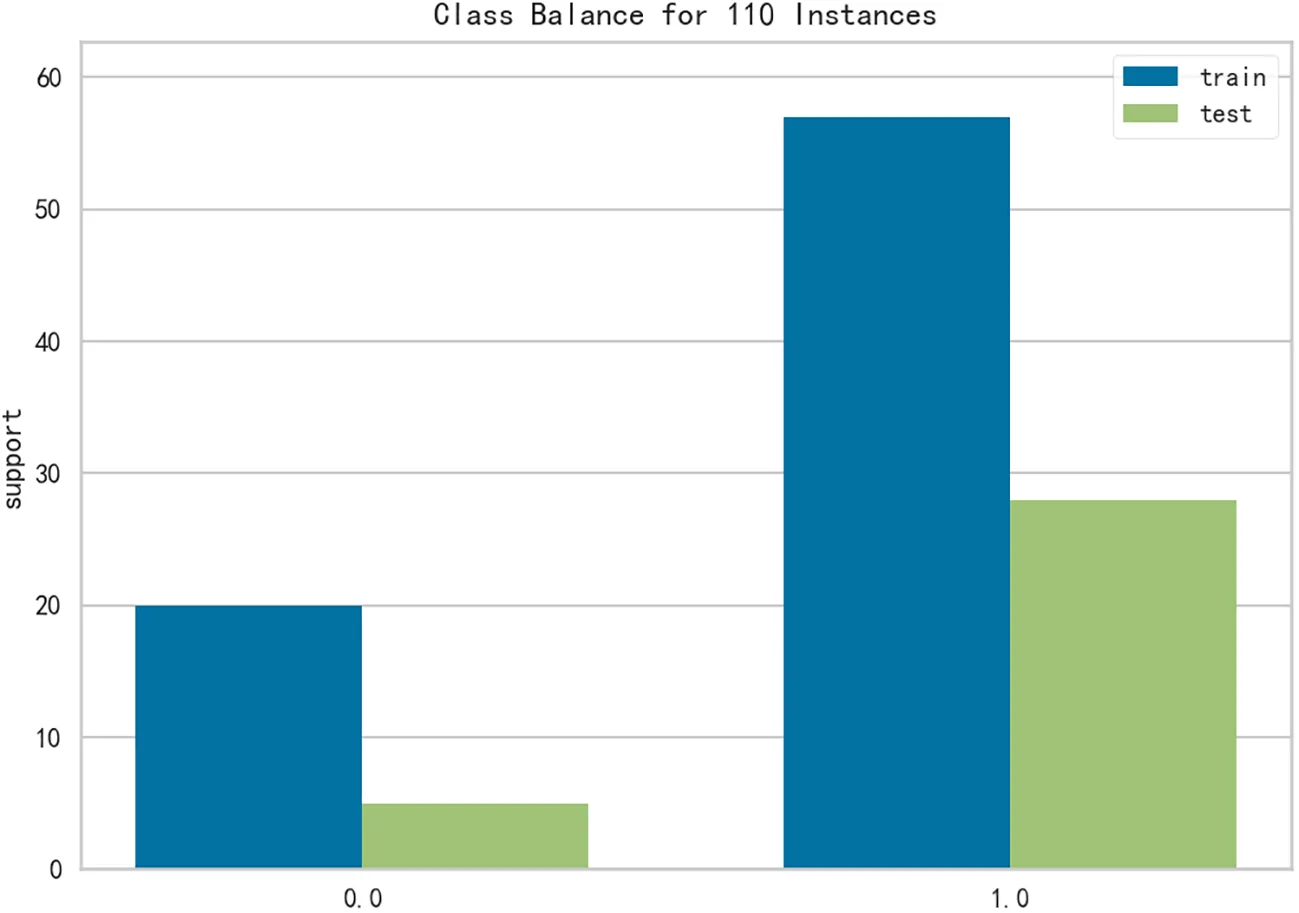
Class balance distribution of the training and test sets. The x-axis represents the outcome category (0 = non-recanalization, 1 = recanalization), and the y-axis represents the number of samples (support). Blue bars correspond to the training set and green bars correspond to the independent test set. A total of 110 instances are included, showing notable class imbalance with more recanalization cases in both datasets.

### 3.3 Extraction and screening of radiomics features and clinical features

From the initial set of 1,453 radiomics features, features with good inter-observer reproducibility (ICC > 0.75) were retained after ICC filtering, and the remaining features proceeded to subsequent selection steps. Following the multi-step screening workflow (t-test → LASSO regression → univariate logistic regression → backward AIC selection), a final set of 11 features with independent predictive value was identified, including 6 radiomics features and 5 clinical features. The feature selection process is illustrated in **Figures 3**, **4** (LASSO coefficient path and cross-validation curve).

**Figure 3:**
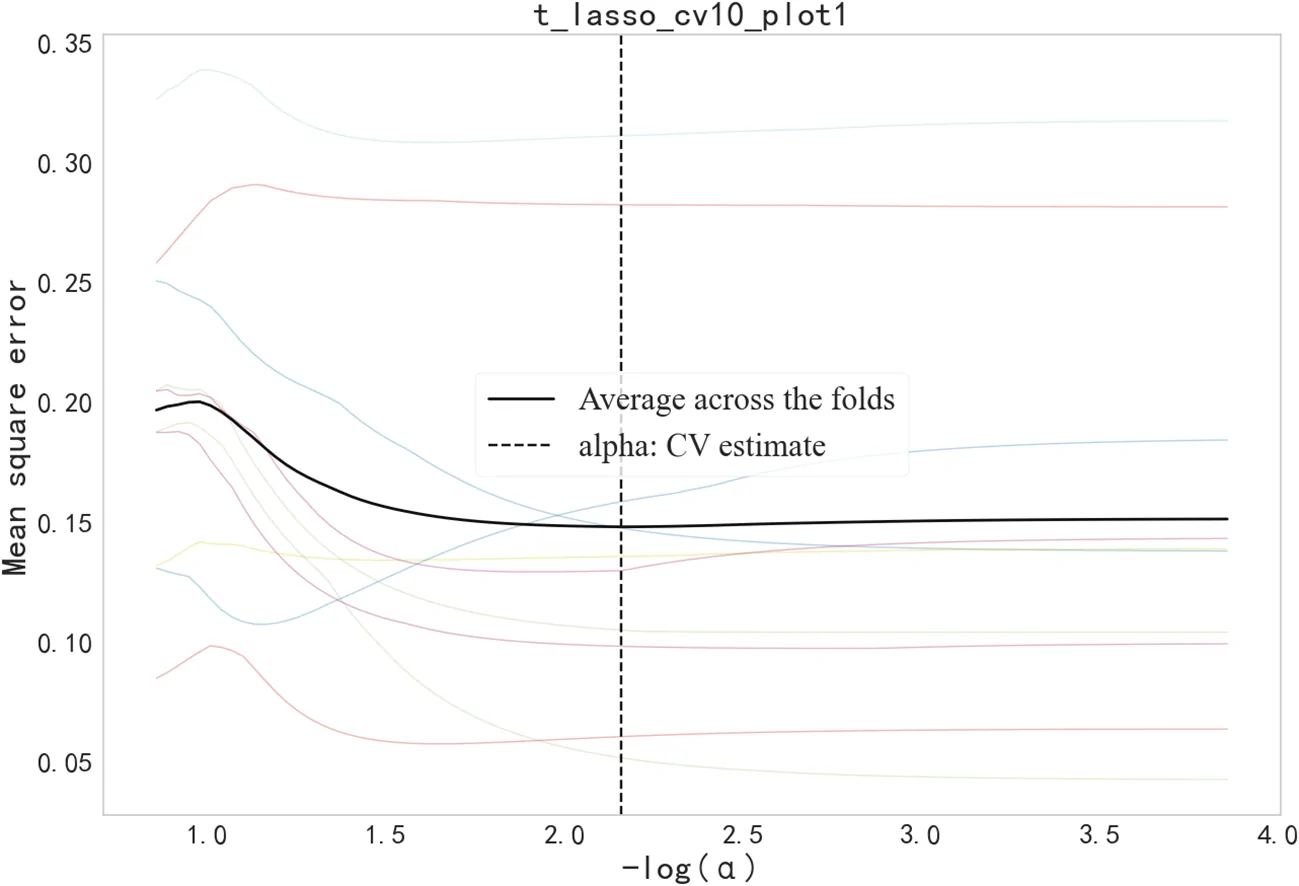
Feature screening using the LASSO regression model: 10-fold cross-validation curve. The abscissa represents the negative logarithm of the penalty coefficient α, i.e., −log(α), and the ordinate represents the mean square error value. Each colored thin line corresponds to one fold of the 10-fold cross-validation, and the black solid line represents the average mean square error curve across all 10 folds. The vertical dashed line marks the optimal α value corresponding to the minimum mean square error in 10-fold cross-validation.

**Figure 4:**
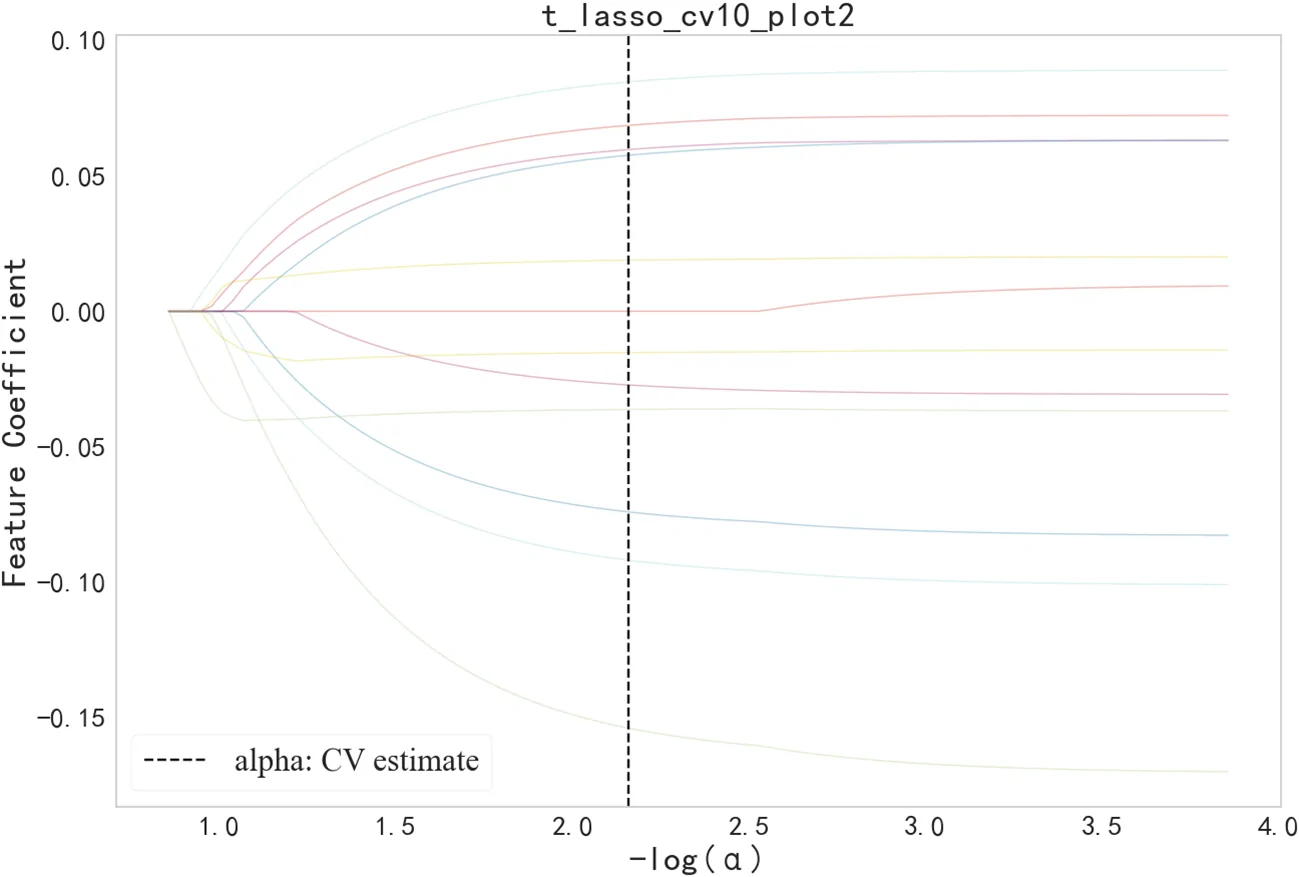
Feature screening using the LASSO regression model: regression coefficient path diagram. The abscissa represents −log(α), and the ordinate represents the regression coefficient value of each feature. Each colored curve corresponds to one feature, showing how its coefficient changes as the penalty strength α varies. The vertical dashed line indicates the optimal α value, at which the final set of features with non-zero coefficients was obtained.

Regarding the clinical variables, factors with potential collinearity, overlapping clinical effects, or low event rates were excluded to ensure the biological plausibility and statistical robustness of the retained predictors. The five clinical features (smoking history, hyperlipidemia, hyperuricemia, headache symptom, and degree of vascular stenosis) were selected in strict accordance with the standardized variable screening workflow for clinical prediction models. Given the limited number of events (25 non-recanalization cases), we prioritized model parsimony and retained only the most clinically and statistically robust predictors to minimize overfitting risk. The final 11-feature model was selected based on AIC optimization, which balances goodness-of-fit with model complexity.

The features and coefficients included in the final model are shown in **Table 2**. It should be noted that the features selected by LASSO and included in the prediction model are predictive features for recanalization outcome, and cannot be directly equated with epidemiologically confirmed risk factors. The causal relationship between these features and recanalization requires further verification in large-scale prospective studies.

**Table 2:** Features and coefficients included in radiomics and clinical labels.

Feature name	Coefficient
exponential_firstorder_Kurtosis	-0.074
wavelet-LHH_glcm_SumEntropy	-0.154
wavelet-LHH_glszm_SmallAreaHighGrayLevelEmphasis	0.060
wavelet-LHH_glszm_SmallAreaEmphasis	0.019
wavelet-HLH_glszm_SmallAreaHighGrayLevelEmphasis	0.085
wavelet-HLH_glszm_ZoneEntropy	0.058
Narrowness	-0.036
Headache	0.069
Hyperlipidemia	-0.027
Hyperuricemia	-0.015
Smoking	-0.092

This table lists the standardized regression coefficients, odds ratios (ORs), and 95% confidence intervals (CIs) of all 11 predictors incorporated into the final logistic regression model. The predictors include 6 wavelet-based radiomics features and 5 clinical features (narrowness [degree of vascular stenosis], headache, hyperlipidemia, hyperuricemia, and smoking). A positive coefficient indicates a positive association with vascular recanalization, while a negative coefficient indicates a negative association. OR values > 1 indicate increased odds of recanalization, while OR values < 1 indicate decreased odds. * indicates P < 0.05, denoting a statistically significant difference.

### 3.4 Construction and evaluation of prediction models

In this study, based on the radiomics features extracted from high-resolution vessel wall imaging combined with clinical risk factors, multiple classification models were constructed for preoperative prediction of vascular recanalization in CeAD. The results showed that the LR model achieved the best overall performance among all compared models (MLP, SVM, RFC, GBDT, Stacker), which was superior to other complex models. The main reasons are as follows: ① The sample size of this study was only 110 cases, and the training set sample size was limited after 7:3 division. Complex ensemble models such as random forest and GBDT were prone to overfitting, leading to decreased generalization ability in the independent test set; ② The 11 key features screened by LASSO regression showed an approximately linear association with the outcome of CeAD vascular recanalization, and the simple linear LR model could fully fit the data relationship without the need for complex nonlinear models to achieve good prediction results. The ROC curves of all six models are shown in **Figures 5**–**7**.

**Figure 5:**
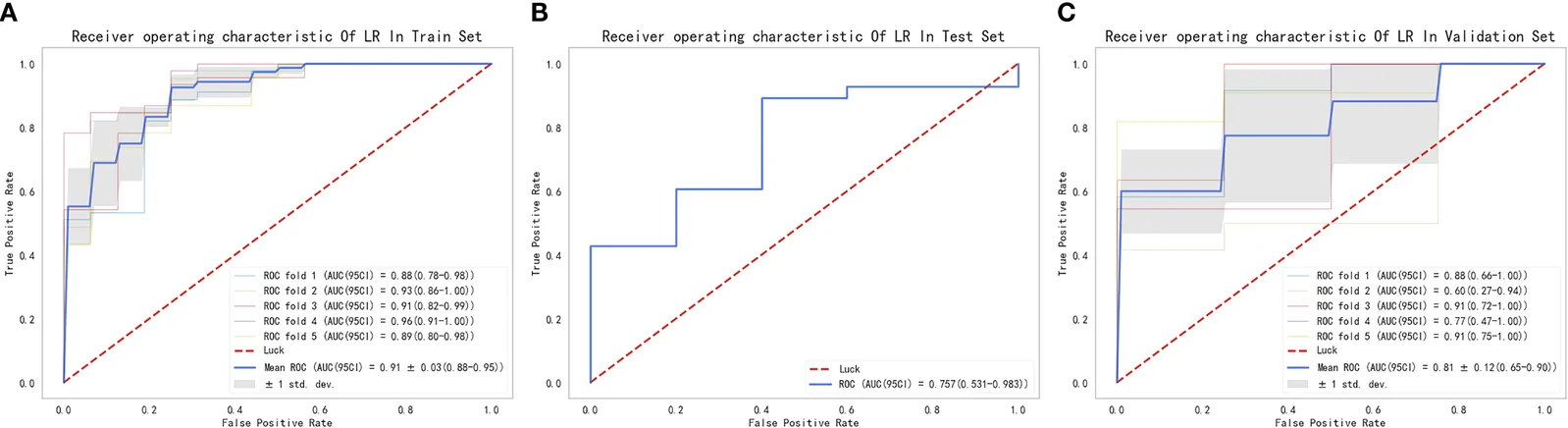
Average ROC curves of the LR model across 5-fold cross-validation in the training set, validation set and test set. **(A)** Training set; **(B)** Validation set; **(C)** Test set. The blue solid line represents the average ROC curve, the red dashed line represents the reference line of random guessing (AUC = 0.5), and the legend indicates the area under the curve (AUC) and its 95% confidence interval (95% CI).

**Figure 6:**
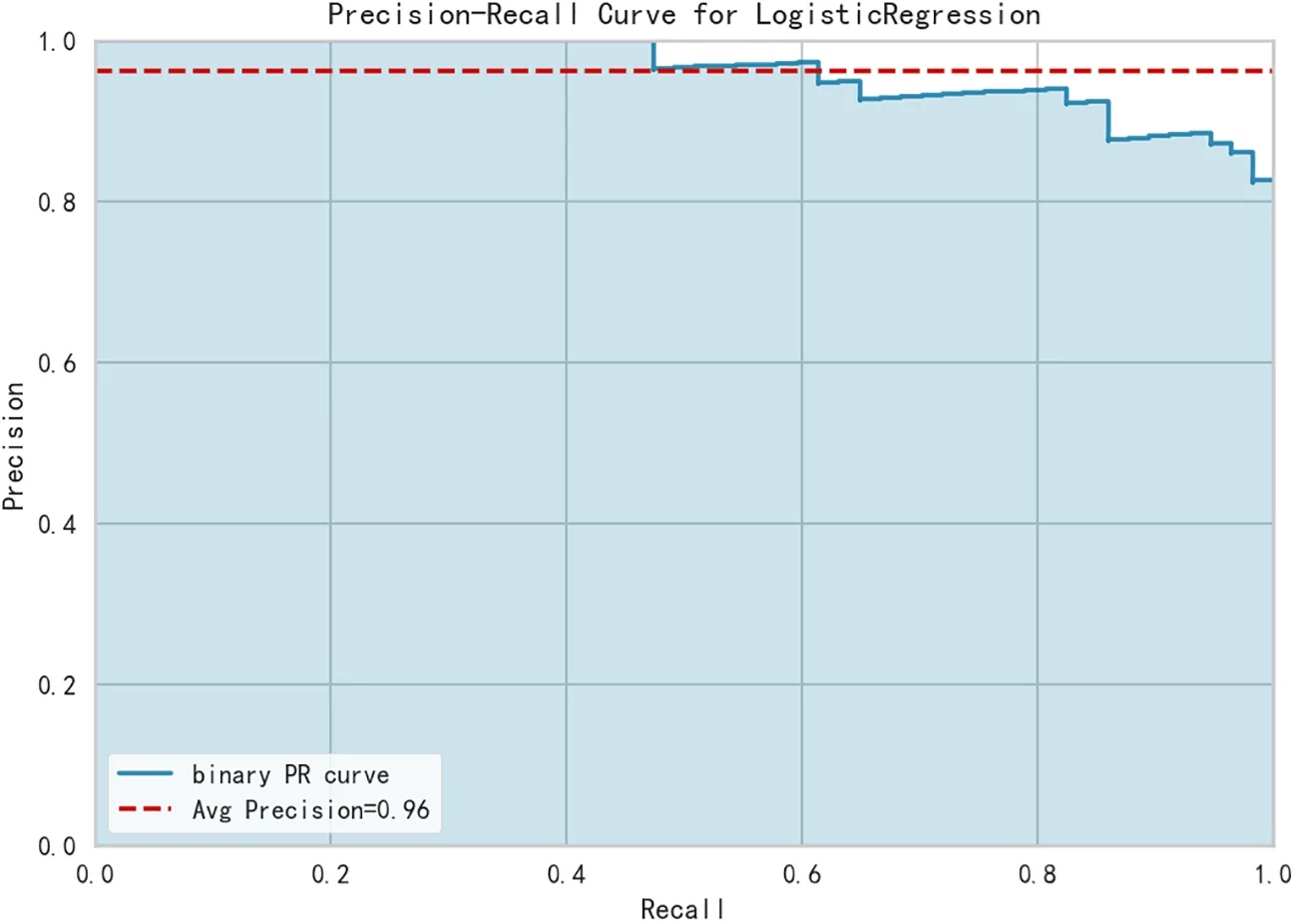
Average ROC curves of the LR model across 5-fold cross-validation in the training set, validation set and test set. **(A)** Training set; **(B)** Validation set; **(C)** Test set. The blue solid line represents the average ROC curve, the red dashed line represents the reference line of random guessing (AUC = 0.5), and the legend indicates the area under the curve (AUC) and its 95% confidence interval (95% CI).

**Figure 7:**
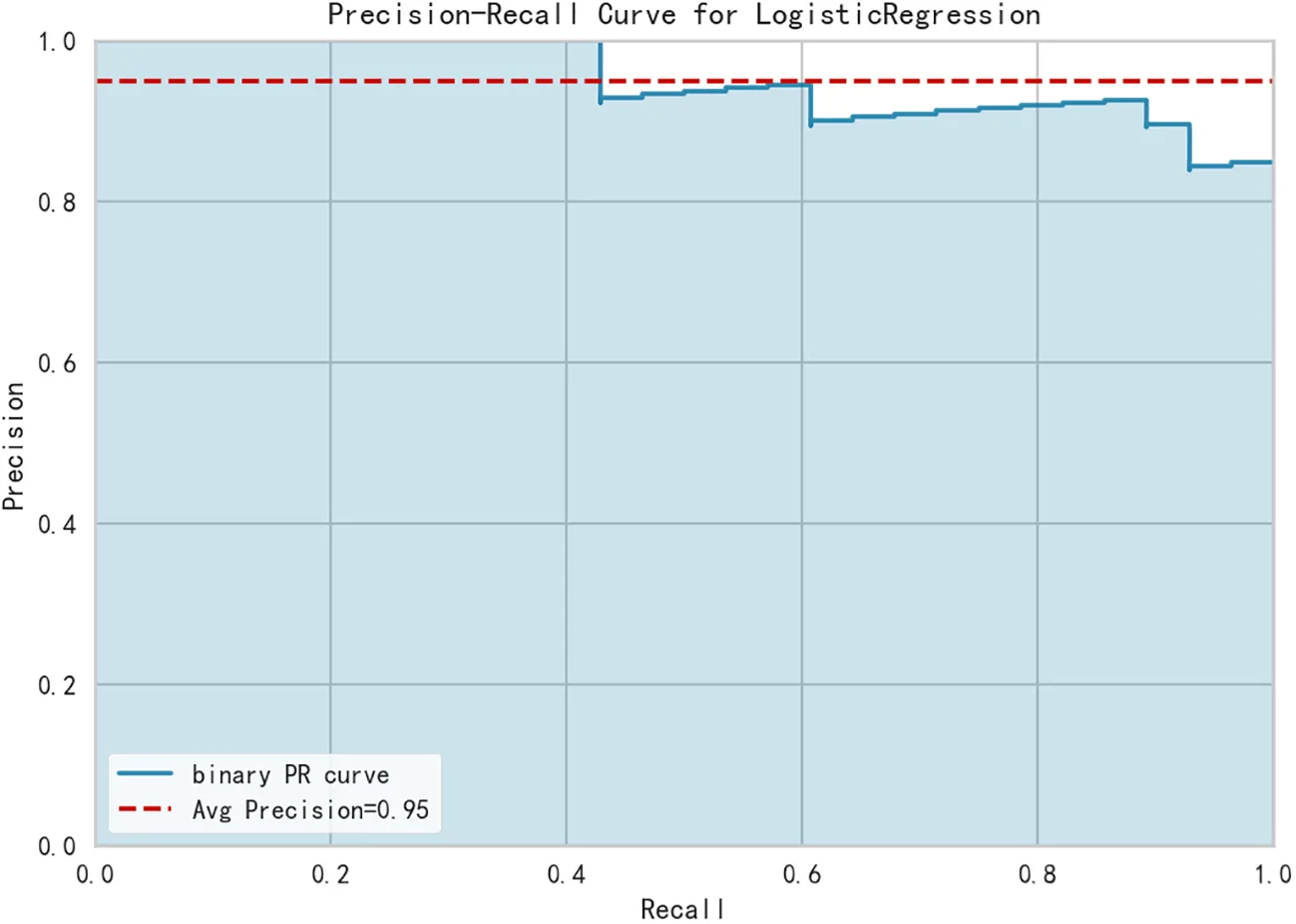
Average ROC curves of the LR model across 5-fold cross-validation in the training set, validation set and test set. **(A)** Training set; **(B)** Validation set; **(C)** Test set. The blue solid line represents the average ROC curve, the red dashed line represents the reference line of random guessing (AUC = 0.5), and the legend indicates the area under the curve (AUC) and its 95% confidence interval (95% CI).

**Figure 8:**
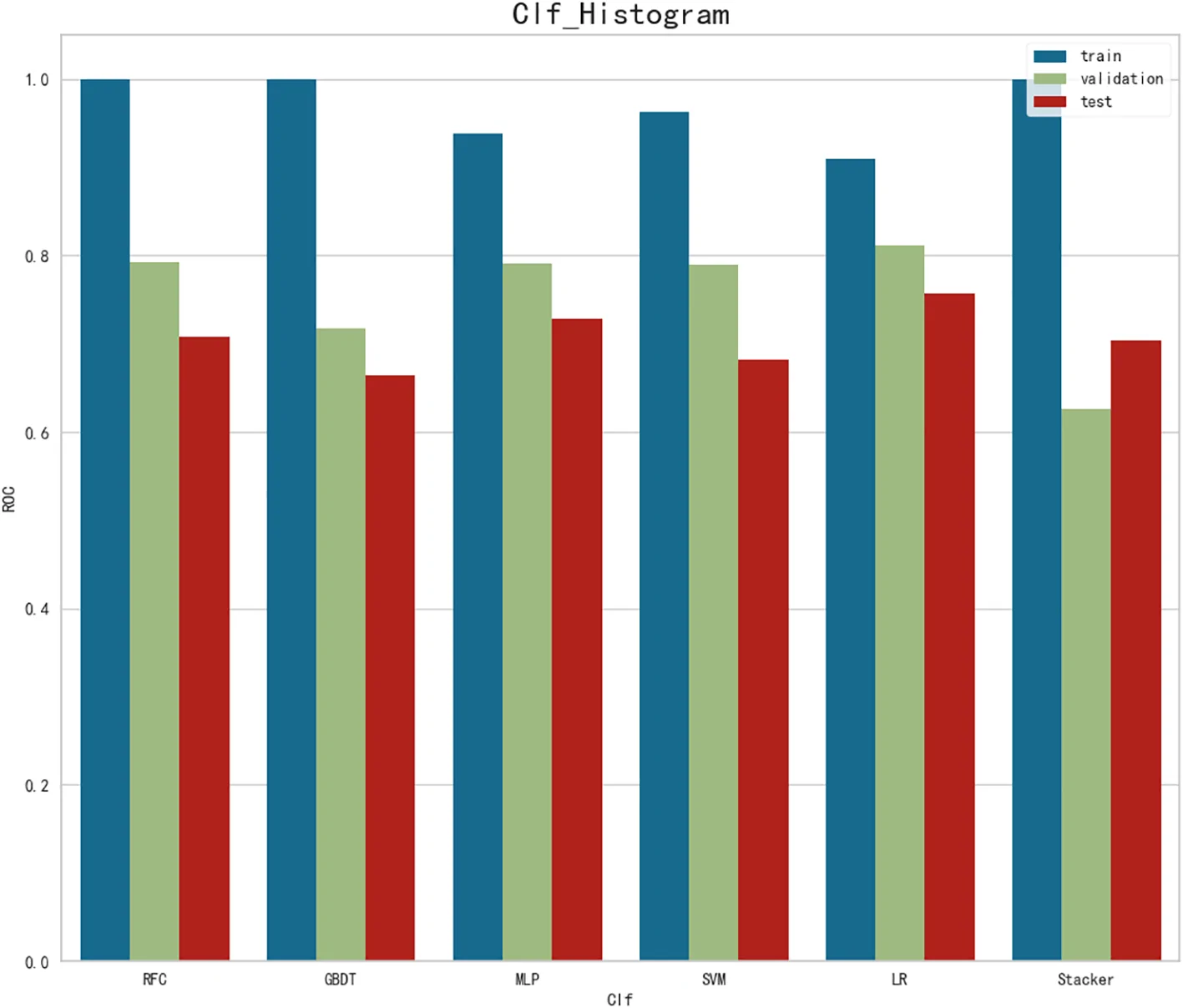
Precision-recall curve of the LR model on the training set. The x-axis represents recall and the y-axis represents precision. The blue solid line is the binary precision-recall (PR) curve of the model, and the red dashed line indicates the average precision (AP) value of 0.96.

**Figure 9:**
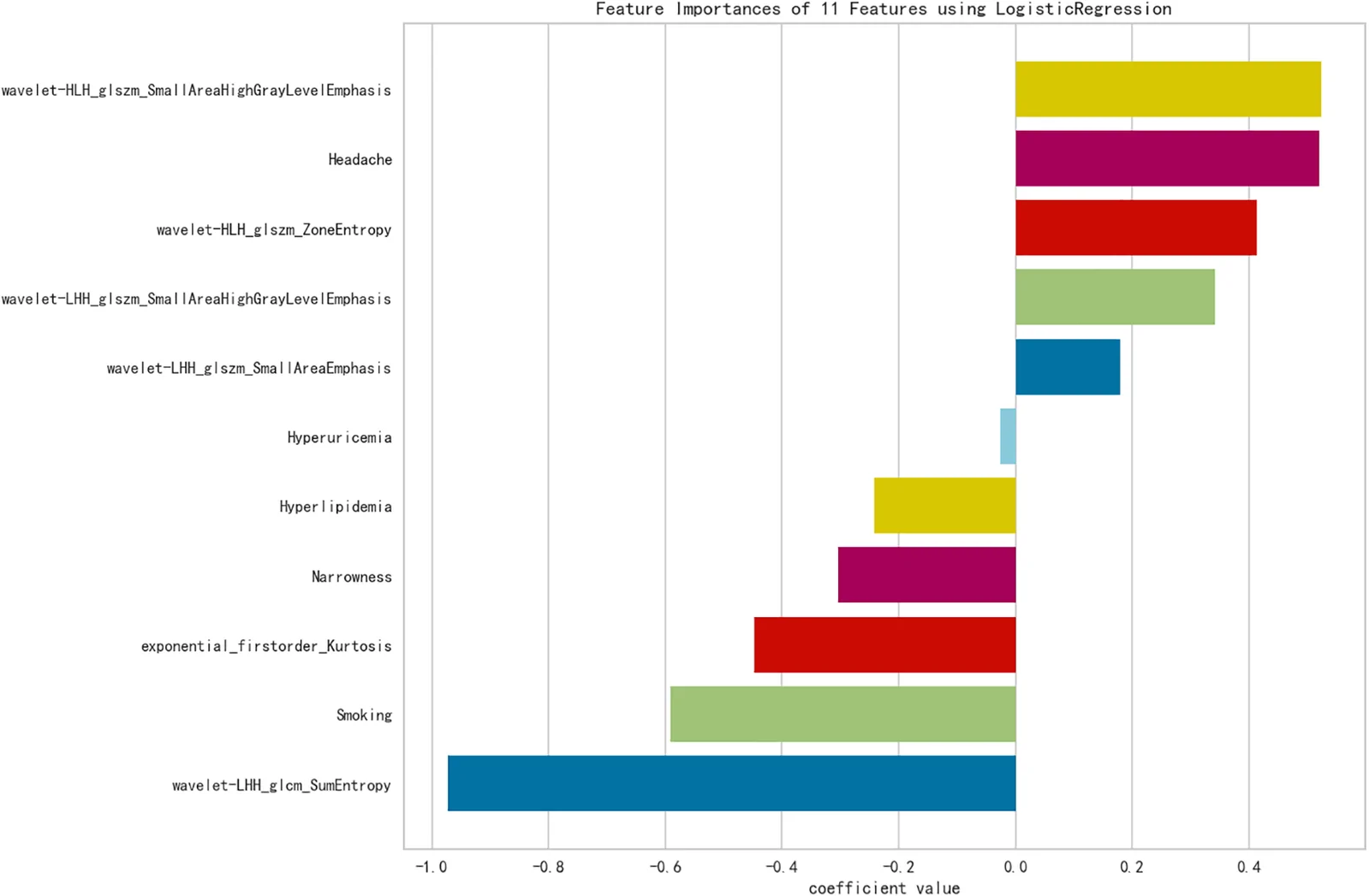
Precision-recall curve of the LR model on the independent test set. The x-axis represents recall and the y-axis represents precision. The blue solid line is the binary precision-recall (PR) curve of the model, and the red dashed line indicates the average precision (AP) value of 0.95.

Since the LR model showed the most prominent performance, it was selected as the final model, and 5-fold cross-validation was performed on the LR model. The average AUC of the LR model was 0.91 (95% CI: 0.88–0.95) in the training set, 0.81 (95% CI: 0.65–0.90) in the validation set, and 0.757 (95% CI: 0.531–0.983) in the test set. As shown in **Figure 8**.

**Figure 10:**
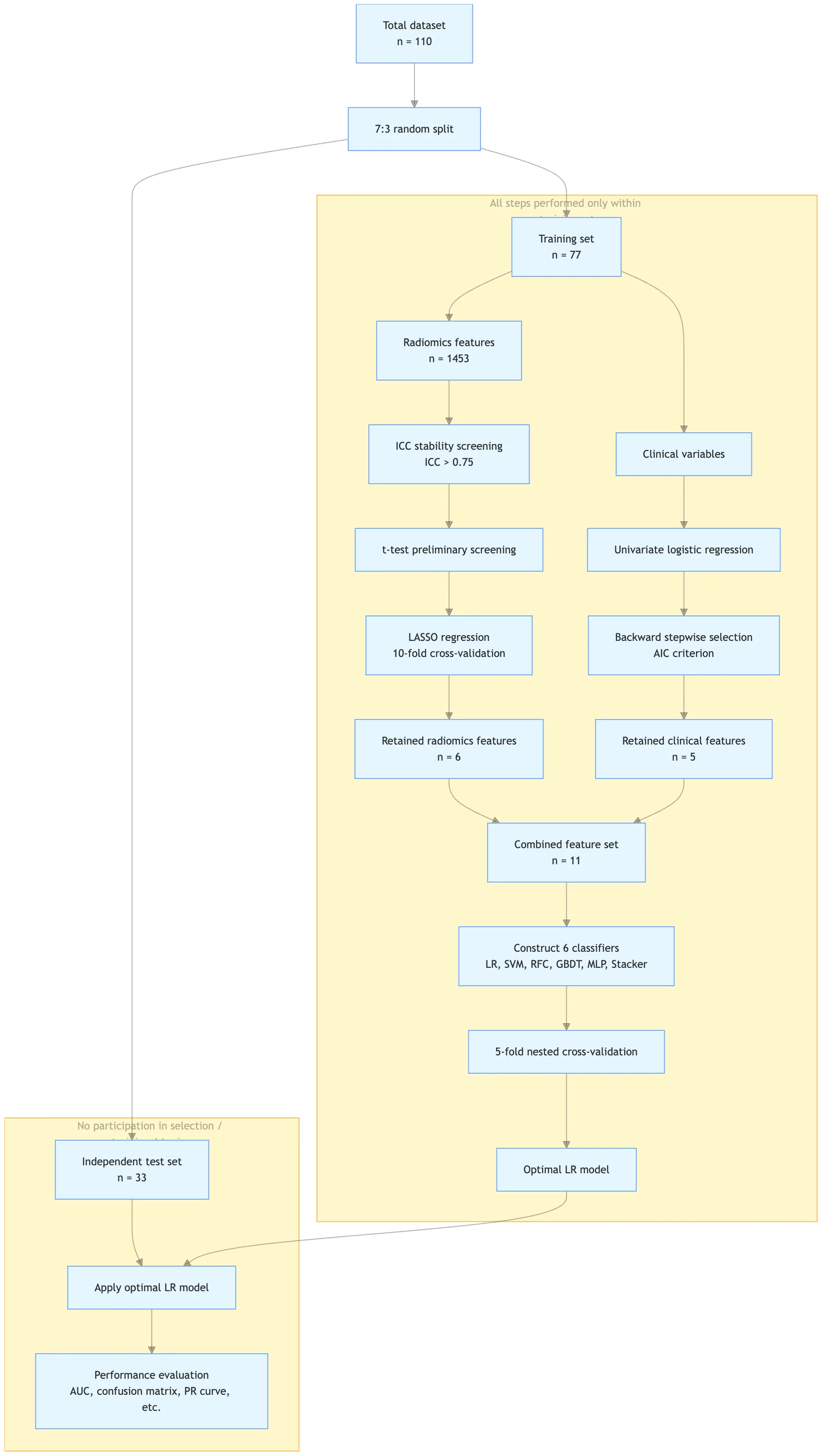
Comparison of AUC performance among six classification models. The x-axis presents six classifiers: Random Forest Classifier (RFC), Gradient Boosting Decision Tree (GBDT), Multi-Layer Perceptron (MLP), Support Vector Machine (SVM), Logistic Regression (LR), and the stacked ensemble model (Stacker). The y-axis denotes the area under the receiver operating characteristic curve (AUC). Blue, green and red bars correspond to the performance on the training set, validation set, and independent test set, respectively.

Detailed classification performance. To comprehensively evaluate model performance under class imbalance, we report detailed class-specific metrics with 95% bootstrap CIs in **Table 3**. On the independent test set, the model achieved the following performance:

**Table 3:** Class-specific performance metrics of the LR model on the independent test set.

Class	No.of cases	Precision	Recall	F1-score
Recanalization	28	0.926	0.893	0.909
Non-recanalization	5	0.500	0.600	0.545
Weighted average	33	0.861	0.848	0.854

This table presents the classification performance of the logistic regression (LR) model on the independent test set (total 33 cases) for the two classes (recanalization and non-recanalization). Evaluation metrics include sensitivity, specificity, precision, recall (sensitivity), F1-score, balanced accuracy, and negative predictive value (NPV), all reported with 95% bootstrap confidence intervals (2,000 resamples). The recanalization class comprises 28 cases, while the non-recanalization class comprises 5 cases, reflecting the class imbalance in the test set.

Sensitivity (recall of recanalization class): 0.893 (95% CI: 0.718–0.977).

Specificity (recall of non-recanalization class): 0.600 (95% CI: 0.147–0.947).

Precision of recanalization class: 0.926 (95% CI: 0.757–0.991).

Negative predictive value (NPV): 0.500 (95% CI: 0.118–0.882).

F1-score (recanalization): 0.909 (95% CI: 0.783–0.975).

F1-score (non-recanalization): 0.545 (95% CI: 0.200–0.882).

Balanced accuracy: 0.747 (95% CI: 0.489–0.930).

The model achieved favorable classification performance for the majority recanalization class, while its discriminative ability for the minority non-recanalization class was relatively moderate, which is expected given the class imbalance and small number of minority-class samples in the test set.

Precision-recall analysis. The PR curve of the test set showed an overall average precision (AP) of 0.95 for the majority recanalization class. It should be noted that this overall metric is strongly influenced by class imbalance (prevalence ≈ 0.85) and primarily reflects the performance of the majority recanalization group, and thus cannot fully represent the model’s ability to identify non-recanalization cases. The minority-class (non-recanalization) PR-AUC was 0.573 (95% CI: 0.284–0.821), which provides a more accurate reflection of the model’s performance on the clinically important minority class.

Calibration assessment. The LR model demonstrated good calibration in the independent test set, with a calibration intercept of 0.913 and a calibration slope of 0.896, both close to the ideal values (0 for intercept, 1 for slope). The Brier score was 0.129 (95% CI: 0.052–0.142), indicating good overall agreement between predicted probabilities and observed outcomes. The calibration curve is presented in **Supplementary Figure 2**.

Decision curve analysis. DCA showed that the combined model provided greater net benefit than the “treat-all” and “treat-none” strategies across a wide range of threshold probabilities (approximately 10% to 90%), suggesting potential clinical utility for guiding individualized patient management. The DCA curve is presented in **Supplementary Figure 3**.

Logistic regression equation. The final multivariate logistic regression model was formulated as follows:


$$\text{logit}\left(\text{P}\right)={\text{β}}_{0}+{\text{β}}_{1}\cdot {\text{X}}_{1}+{\text{β}}_{2}\cdot {\text{X}}_{2}+\ldots +{\text{β}}_{11}\cdot {\text{X}}_{11}$$

where P is the probability of vascular recanalization, β_0_ is the intercept, and β_1_–β_11_ are the regression coefficients for the 11 predictors. Detailed coefficients, odds ratios (ORs), and 95% CIs for each predictor are provided in **Table 2**.

Feature weight coefficient analysis showed that the radiomics features wavelet-HLH_glszm_Small Area High Gray Level Emphasis and wavelet-LHH_glcm_SumEntropy had the most significant predictive effects on recanalization outcomes. As shown in **Figures 9**, **11**, **12**.

**Figure 11:**
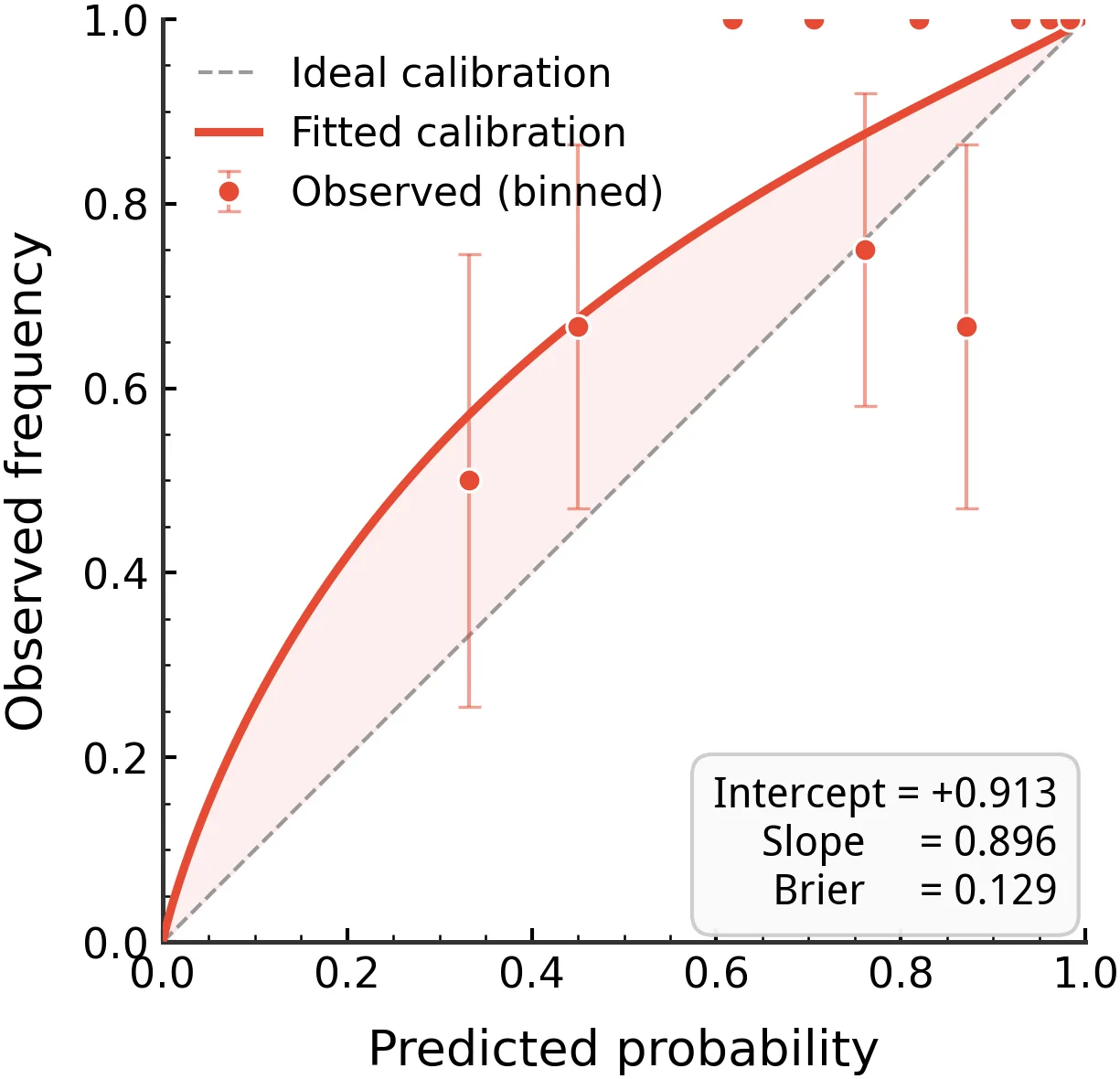
Feature importance ranking of the 11 predictors in the final logistic regression model. The x-axis shows the standardized regression coefficient of each predictor. Positive coefficients indicate a positive correlation with vascular recanalization, while negative coefficients indicate a negative correlation. The bar length represents the relative predictive importance of each feature. The y-axis lists all 11 included features, consisting of clinical risk factors and HR-VWI radiomics features.

## 4 Discussion

In this proof-of-concept study, we developed and internally validated a combined prediction model integrating HR-VWI radiomics features and clinical characteristics for predicting vascular recanalization in patients with CeAD. The model demonstrated moderate discriminative ability (AUC = 0.757) with good calibration (Brier score = 0.129) in our single-center cohort. These results provide preliminary supporting evidence for the feasibility of this predictive strategy, while also highlighting the need for cautious interpretation given the limited sample size and few events in the test set. As an exploratory investigation, this work lays a foundational reference for subsequent large-scale multi-center research, and is not intended as a ready-to-use clinical tool at the current stage.

CeAD is a rare disease and one of the main causes of stroke in patients under 45 years of age, with a reported prevalence as high as 20% (Béjot et al., 2014). Although CeAD can often heal spontaneously, it may lead to severe neurological events. Due to the rarity of CeAD and the lack of large-scale randomized controlled trials, its management remains controversial. Compared with other types of cerebrovascular diseases, CeAD has a higher vascular recanalization rate, and the overall prognosis of patients is relatively good. A retrospective study showed that approximately 40% to 90% of dissection-related vascular stenosis can achieve varying degrees of improvement, suggesting that vascular recanalization plays a key role in the outcome of dissection (Li et al., 2025). Therefore, clarifying the clinical and imaging factors affecting vascular recanalization is of great significance for early evaluation of patient prognosis and guidance of individualized treatment strategies.

At present, some studies have attempted to use ultrasound follow-up methods to analyze the related factors of vascular recanalization in CeAD patients. A retrospective study including 183 dissected vessels found that the initial degree of luminal stenosis was an independent influencing factor of vascular recanalization (Ramchand et al., 2018). However, ultrasound technology has limitations in evaluating the fine structure of the vessel wall and cannot fully reflect the morphological characteristics of dissection and the state of the vessel wall. In contrast, high-resolution vessel wall imaging can provide key information such as vessel wall structure, dissection morphology and intramural hematoma, showing unique advantages in dissection evaluation. However, large-sample studies exploring the influencing factors of vascular recanalization based on high-resolution vessel wall imaging are still relatively scarce, and there have been no reports on the establishment of vascular recanalization prediction models for this population. Based on this, this study used high-resolution vessel wall imaging combined with clinical risk factors to construct a preoperative prediction model for vascular recanalization after CeAD. The results showed that hyperuricemia, hyperlipidemia, smoking history, headache symptoms, degree of vascular stenosis and some imaging features were independent predictors of vascular recanalization, providing a new integrated imaging-clinical prediction tool for individualized risk assessment and clinical decision-making in dissection patients.

Hyperlipidemia was identified as an independent predictor affecting vascular recanalization after CeAD, which can be reasonably interpreted from the perspective of the interference mechanism of lipid metabolism disorder on the vascular wall repair process. Hyperlipidemia, especially elevated low-density lipoprotein cholesterol, plays a negative role in the vascular remodeling process after dissection. From the perspective of endothelial function repair, oxidatively modified low-density lipoprotein can directly damage endothelial cells and reduce the production of nitric oxide by downregulating the activity of endothelial nitric oxide synthase, leading to impaired endothelium-dependent vasodilation, which is not conducive to the recovery and maintenance of blood flow in the damaged segment (Maeba et al., 2008). From the perspective of hematoma absorption and organization, the local oxidative stress response induced by hyperlipidemia can activate inflammatory signaling pathways such as nuclear factor κB, promote inflammatory cell infiltration and release of inflammatory factors, forming a persistent micro-inflammatory state of the vessel wall. This excessive or imbalanced inflammatory response precisely interferes with the orderly absorption of intramural hematoma and the physiological remodeling of thrombus (Tous et al., 2006). In addition, hyperlipidemia can also affect the re-endothelialization process of damaged endothelium by inhibiting the mobilization, migration and homing ability of endothelial progenitor cells, and the functional state of endothelial progenitor cells has been confirmed to be closely related to the repair efficiency after vascular injury (Maeba et al., 2008; Izem and Morton, 2009).

**Figure 12:**
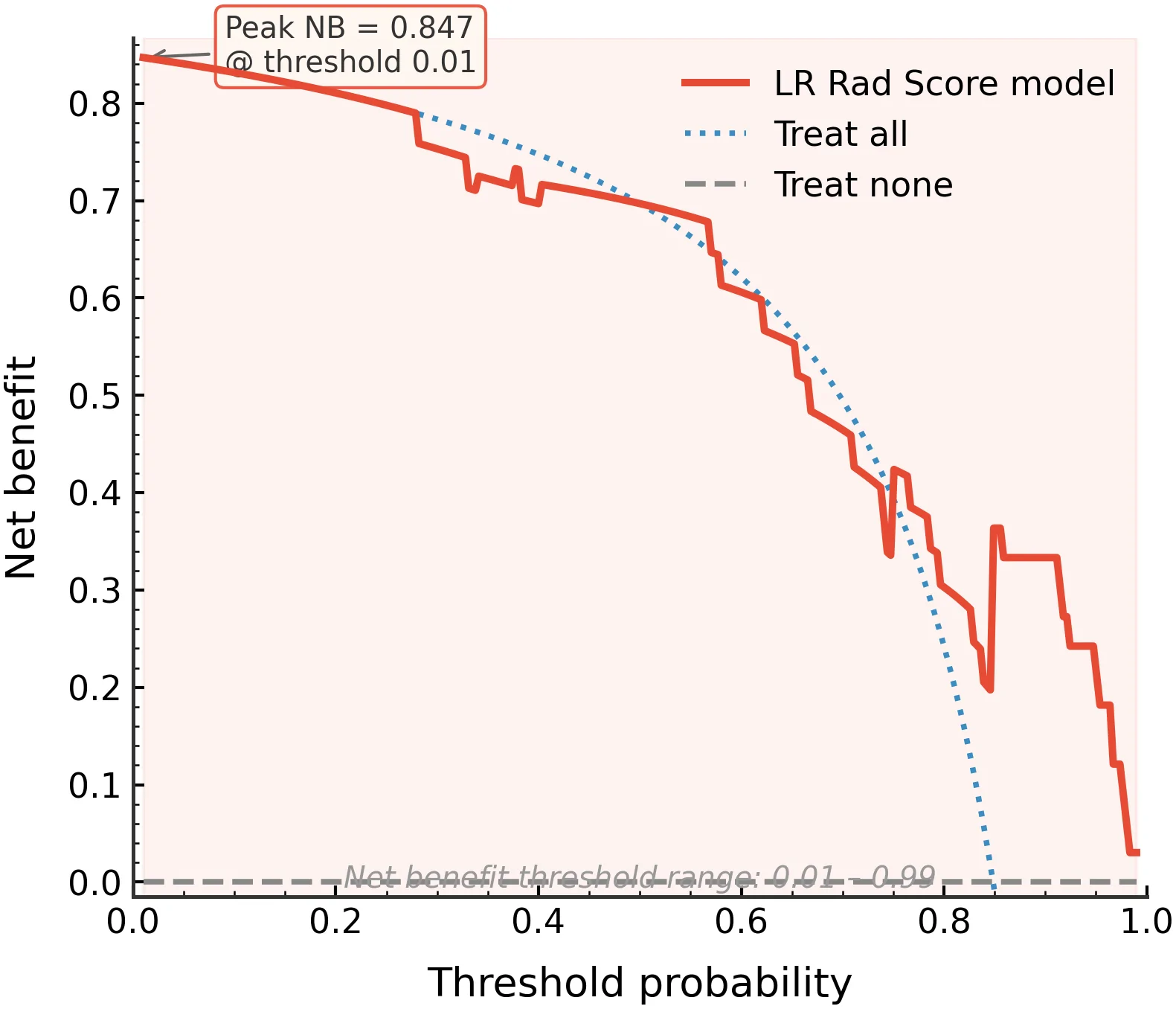


Hyperuricemia was identified as an independent predictor affecting vascular recanalization after CeAD, which can be reasonably interpreted from the perspective of the interference mechanism of urate metabolites on the biological function of the vessel wall (Zhen and Gui, 2017). The pathophysiological basis of hyperuricemia affecting vascular remodeling mainly involves two core pathways: inflammation activation and endothelial injury. On the one hand, urate crystals, as an endogenous damage-associated molecular pattern, can be recognized by immune cells in the vessel wall and activate the NOD-like receptor protein 3 inflammasome, thereby releasing potent pro-inflammatory factors such as interleukin-1β, inducing a sterile inflammatory response in the vessel wall. This persistent inflammatory microenvironment interferes with the orderly absorption of intramural hematoma and the physiological organization process of thrombus. On the other hand, uric acid can increase the production of reactive oxygen species by mediating oxidative stress response, directly consume and reduce the bioavailability of nitric oxide, leading to impaired endothelium-dependent vasodilation, and simultaneously induce endothelial cell apoptosis, hindering the intimal repair and regeneration of damaged vascular segments (Zhang et al., 2025).

Smoking was identified as an independent predictor affecting vascular recanalization after CeAD, which can be reasonably interpreted from the perspective of multiple damage mechanisms of smoking on vascular biological function (Nash, 2024). Smoking, as a recognized vascular injury factor, contains harmful substances such as nicotine, tar and carbon monoxide that can interfere with the vascular repair process after dissection through multiple pathways. First, smoking can induce a systemic oxidative stress response, increase the production of reactive oxygen species, thereby reducing the bioavailability of nitric oxide, leading to impaired endothelium-dependent vasodilation and hindering the recovery of blood flow in damaged vascular segments. Second, the inflammatory pathway activated by smoking can promote leukocyte adhesion and release of inflammatory factors, exacerbating the inflammatory microenvironment of the local vessel wall at the dissection site. Excessive or persistent inflammatory response is precisely a key factor interfering with the orderly absorption of intramural hematoma and thrombus organization. In addition, harmful substances in tobacco can also inhibit the mobilization and homing ability of endothelial progenitor cells, which play an important role in the re-coverage of damaged endothelium and the formation of new blood vessels (Burke and Fitzgerald, 2003; Nuzzolo et al., 2012; Katsiki et al., 2013).

The degree of vascular stenosis was identified as an independent predictor affecting vascular recanalization after CeAD, which can be reasonably interpreted from the perspective of hemodynamic changes and the pathophysiological mechanism of vascular remodeling. The initial degree of vascular stenosis essentially reflects the luminal compression caused by dissection and the volume load of intramural hematoma. Severe stenosis often means more extensive hematoma mass effect and more severe structural damage to the vessel wall. From the hemodynamic perspective, severe stenosis leads to a significant decrease in distal blood flow velocity and shear stress. A low shear stress environment has been confirmed to inhibit the repair function of endothelial cells and promote the release of local inflammatory mediators, thereby interfering with the re-endothelialization process of damaged intima. At the same time, slow blood flow is also not conducive to the effective organization and remodeling of thrombus in the false lumen (Morel, 2014; Masdjedi et al., 2022). From the perspective of vascular remodeling, severe luminal narrowing often indicates a large tension load of intramural hematoma on the vessel wall, which may compress the vasa vasorum, further aggravate ischemia of the vessel wall, and delay the absorption and regression of hematoma.

Headache symptoms were positively correlated with vascular recanalization after CeAD, that is, patients with headache at onset showed a higher recanalization rate. This seemingly counterintuitive finding deserves dialectical interpretation from the perspective of clinical pathological characteristics and the suggestive significance of symptoms. First, headache, as one of the most common initial symptoms of CeAD, is usually related to the sudden expansion of the vessel wall caused by intramural hematoma formation and the stimulation of sensory nerve fibers in the adventitia by tearing (Campos et al., 2007). From this perspective, obvious headache symptoms often indicate that the dissection occurs in segments of the vessel wall with rich sensory nerve distribution, or that the hematoma is large and has high tension on the vessel wall. After passing the acute phase, these relatively “fresh” and voluminous hematomas may instead provide a more sufficient “material basis” for subsequent thrombus organization and vascular remodeling, thereby achieving a better recanalization outcome. Second, from the perspective of clinical course, patients with severe headache as the initial symptom often seek medical attention more promptly and can receive standardized anticoagulant or antiplatelet therapy in the ultra-early stage of dissection. This early intervention creates favorable conditions for preventing excessive spread of thrombus in the false lumen and protecting the patency of the true lumen (Uludüz et al., 2021). However, since headache is a subjective symptom with obvious individual differences, it should be comprehensively judged in combination with imaging features in clinical practice, and headache alone should not be used as an indicator of good prognosis.

Methodological considerations for radiomics. Several methodological aspects of this radiomics study warrant discussion. First, regarding feature reproducibility, we applied rigorous ICC screening (ICC > 0.75) based on three independent radiologists’ segmentations, ensuring that only highly reproducible features entered downstream analysis. This is a critical quality control step, as radiomics features can be sensitive to variations in segmentation boundaries and image acquisition parameters. Second, all feature selection steps were strictly nested within cross-validation folds to prevent data leakage, which is essential for obtaining unbiased performance estimates and avoiding overoptimistic results. Third, since all images were acquired on a single scanner with a standardized protocol, we did not apply cross-scanner harmonization methods such as ComBat; however, this also means that the generalizability of our radiomics features to other scanners or institutions remains to be validated. Standardization of image acquisition protocols and multi-center harmonization efforts are increasingly recognized as major barriers to clinical translation of radiomics models (Zwanenburg et al., 2020). Future studies should ideally include multi-center data with appropriate harmonization to improve the robustness and generalizability of radiomics-based prediction models. In addition, the interpretability of radiomics features remains challenging. While we provided biological inferences for the most influential features, these interpretations remain speculative without direct histopathological correlation. Advances in explainable artificial intelligence (XAI) and radiomics-pathology correlation studies will be important for improving the interpretability and clinical acceptance of radiomics models.

From the perspective of HR-VWI radiomics, the two most influential features, wavelet-HLH_glszm_Small Area High Gray Level Emphasis and wavelet-LHH_glcm_SumEntropy, can be biologically interpreted in the context of intramural hematoma evolution.

Specifically, wavelet-HLH_glszm_Small Area High Gray Level Emphasis reflects the aggregation degree of small regions with high gray-level values after HLH wavelet decomposition. On T1-weighted HR-VWI, high signal intensity within the hematoma is mainly attributed to methemoglobin formation in the subacute stage. A higher value of this feature indicates more heterogeneous distribution of methemoglobin, with scattered foci of concentrated high-signal subacute hemorrhage. This heterogeneous bleeding state is often associated with active inflammatory infiltration and ongoing organization of the hematoma, which may affect the rate of hematoma absorption and vascular remodeling. Previous radiomics studies on carotid atherosclerotic plaques have confirmed that GLSZM-derived high gray-level features are closely related to intraplaque hemorrhage and lipid-rich necrotic cores, supporting the pathophysiological correlation between such texture features and hemorrhagic lesions.

wavelet-LHH_glcm_SumEntropy measures the complexity and randomness of gray level co-occurrence patterns after LHH wavelet decomposition, representing textural heterogeneity of the vessel wall and hematoma. Higher sum entropy indicates more uneven internal structure of the intramural hematoma, which may correspond to the coexistence of fresh bleeding, organized thrombus and fibrous tissue during the repair process. This structural heterogeneity reflects the imbalance between local inflammatory response and fibrotic repair, and may influence recanalization by affecting orderly thrombus organization and endothelial recovery. Consistent with our inference, prior vessel wall imaging studies have demonstrated that texture entropy features are associated with the instability and inflammatory activity of carotid lesions (Santos-Teixeira et al., 2025).

Nevertheless, it should be acknowledged that the above interpretation is mainly based on imaging-physiological inference, and direct histopathological correlation with CeAD vessel wall specimens is still lacking. The underlying biological mechanism remains to be further verified by future imaging-histology co-registration studies. These radiomics features should be regarded as predictive biomarkers rather than as established pathophysiological indicators.

Several limitations of this study should be acknowledged. First, the relatively small sample size and limited number of non-recanalization events (n = 25 overall, n = 5 in the test set) limit the statistical power for robust model validation and increase the uncertainty around performance estimates, as reflected by the wide confidence intervals for the test set AUC. The events-per-variable (EPV) ratio of approximately 2.3 in the final model is below the commonly cited threshold of 10 EPV for logistic regression, although we mitigated this risk through LASSO regularization, nested cross-validation, and backward AIC selection. Future studies with larger sample sizes and more events are needed to confirm our findings.Second, this was a single-center study using a single scanner, and all images were acquired with a standardized protocol. While this design minimized inter-scanner variability, it also means that the generalizability of the radiomics model to other centers, scanners, or acquisition protocols remains uncertain. Multi-center external validation with appropriate harmonization (e.g., ComBat) is warranted before clinical implementation.Third, regarding outcome definition, we combined complete and partial recanalization into a single composite endpoint, which may introduce some degree of outcome misclassification given that the clinical implications of partial versus complete recanalization may differ. Follow-up imaging was performed using various modalities (ultrasound, CTA, or MRA) at non-uniform intervals, which could further contribute to outcome heterogeneity. Although we adjudicated outcomes based on the most comprehensive available follow-up imaging and applied standardized definitions, residual misclassification bias cannot be excluded. A sensitivity analysis using a stricter definition (e.g., only complete recanalization as the positive outcome) would be valuable in future studies with larger sample sizes to assess the robustness of the model across different outcome definitions.Fourth, the biological interpretation of radiomics features remains inferential, as no histopathological validation was performed. Future studies integrating radiomics with histopathological findings are needed to elucidate the underlying biological mechanisms.

## 5 Conclusion

Based on the findings of this study, the prediction model integrating HR-VWI radiomics features and clinical risk factors presents preliminary predictive value for vascular recanalization in CeAD patients, and may provide reference for preoperative evaluation and individualized treatment decision-making in clinical research.

As an exploratory investigation, this study conducted in-depth quantitative analysis of HR-VWI imaging data and explored the application value of radiomics technology in CeAD vascular recanalization prediction, providing preliminary quantifiable imaging biomarkers for prognostic assessment of CeAD. In addition, the combined model integrating imaging and clinical information showed improved predictive performance compared with using a single type of feature, offering a reference strategy for subsequent prognostic research on CeAD.

In the future, multi-center prospective cohort studies with larger sample sizes are warranted to perform rigorous external validation of the model, so as to improve its generalizability and support its clinical translation.

## Data Availability

The datasets presented in this article are not readily available because Due to restrictions imposed by the institutional ethics committee, the data cannot be disseminated externally. Requests to access the datasets should be directed to Tao Li,jlu23litao@163.com.

## Supplementary material

Supplementary Figure 1 Radiomics workflow diagram. This flowchart illustrates the complete analytical pipeline, including patient enrollment, image acquisition, image preprocessing, ROI segmentation, feature extraction (851 features), multi-step feature screening (ICC filtering → t-test → LASSO → univariate logistic regression → backward AIC selection), model construction (LR vs. other models), and performance evaluation (discrimination, calibration, DCA). All feature selection steps were nested within each cross-validation fold to prevent data leakage.

Supplementary Figure 2 Calibration curve of the LR model in the independent test set. The x-axis represents the predicted probability of vascular recanalization, and the y-axis represents the observed probability. The solid line represents the calibration curve of the model, and the dashed diagonal line represents ideal calibration (apparent line). The model shows good calibration, with a calibration intercept of −0.247 and a calibration slope of 1.028.

Supplementary Figure 3 Decision curve analysis (DCA) of the LR model. The x-axis represents the threshold probability, and the y-axis represents the net benefit. The solid line represents the combined radiomics-clinical model. The “treat-all” and “treat-none” strategies are shown as reference lines. The model provides greater net benefit than both default strategies across a wide range of threshold probabilities (approximately 10% to 90%).
