# Supplementary material for: Development of a preoperative prediction model for vascular recanalization in cervical artery dissection based on HR-VWI radiomics features combined with clinical risk factors
Source: Front Physiol. 2026 Sep 14;17:1886681. doi: 10.3389/fphys.2026.1886681 (PMC13616681; doi:10.3389/fphys.2026.1886681)
Supplement: Supplementary file 1 [file Table1.docx]

Table1 Comparison of baseline characteristics between patients in the recanalization group and non-recanalization group

| **Item** | **Recanalization group (n=85)** | **Non-recanalization group (n=25)** | **t/χ²/Z value** | **P value** |
| --- | --- | --- | --- | --- |
| Age (years, xˉ±s) | 45.52 ± 13.10 | 48.84 ± 13.54 | -1.107 | 0.271 |
| Male [n (%)] | 54 (63.53) | 22 (88.0) | 5.6 | 0.018* |
| Hypertension [n (%)] | 29 (34.12) | 11 (44.0) | 0.80 | 0.372 |
| Diabetes [n (%)] | 6 (7.06) | 3 (12.00) | 1.07 | 0.300 |
| History of stroke [n (%)] | 42 (49.41) | 16 (64.00) | 1.65 | 0.198 |
| Migraine [n (%)] | 36 (42.35) | 5 (20.0) | 4.13 | 0.042* |
| Coronary heart disease [n (%)] | 9 (10.59) | 3 (12.00) | 0.04 | 0.842 |
| Hyperlipidemia [n (%)] | 26 (30.59) | 12 (48.00) | 2.42 | 0.120 |
| Smoking [n (%)] | 32 (37.65) | 17 (68.00) | 7.19 | 0.007* |
| Drinking [n (%)] | 20 (23.53) | 11 (44.00) | 3.45 | 0.063 |
| Trauma history [n (%)] | 7 (8.24) | 0 (0.00) | 0.71 | 0.400 |
| Family history [n (%)] | 20 (23.53) | 2 (8.00) | 0.05 | 0.829 |
| Infection history [n (%)] | 21 (27.06) | 2 (8.00) | 4.02 | 0.045* |
| Time from symptom onset to admission [days, M(P25, P75)] | 10 (7, 12.67) | 12 (8.9, 16) | -0.984 | 0.325 |
| Intimal flap [n (%)] | 19 (22.35) | 5 (20.00) | 0.06 | 0.804 |
| Double lumen sign [n (%)] | 40 (47.06) | 9 (36.00) | 0.51 | 0.476 |
| Intraluminal thrombus [n (%)] | 21 (24.71) | 5 (20.00) | 0.25 | 0.615 |
| Dissection length [cm, M(P25, P75)] | 3.2 (1.6, 4.7) | 3 (1.25, 5.25) | -0.168 | 0.867 |
| Signal type of intramural hematoma [n (%)] | - | - | 4.146 | 0.126 |
| Iso-signal | 18 (21.2) | 3 (12.0) | - | - |
| Slightly high signal | 20 (23.5) | 8 (32.0) | - | - |
| High signal | 47 (55.3) | 14 (56.0) | - | - |
| Degree of vascular stenosis [n (%)] | - | - | 18.852 | 0.001* |
| Mild | 22 (25.9) | 1 (4.0) | - | - |
| Moderate | 18 (21.2) | 5 (20.0) | - | - |
| Severe | 28 (32.9) | 8 (32.0) | - | - |
| Occlusion | 17 (20.0) | 11 (44.0) | - | - |
| Antiplatelet therapy [n (%)] | 77 (91.6) | 22 (88) | 0.30 | 0.584 |
| Anticoagulant therapy [n (%)] | 30 (35.29) | 5 (20.00) | 2.08 | 0.149 |

*: P < 0.05; -: No statistical value

Table2 Features and coefficients included in radiomics and clinical labels

| **Feature name** | **Coefficient** |
| --- | --- |
| exponential_firstorder_Kurtosis | -0.074 |
| wavelet-LHH_glcm_SumEntropy | -0.154 |
| wavelet-LHH_glszm_SmallAreaHighGrayLevelEmphasis | 0.060 |
| wavelet-LHH_glszm_SmallAreaEmphasis | 0.019 |
| wavelet-HLH_glszm_SmallAreaHighGrayLevelEmphasis | 0.085 |
| wavelet-HLH_glszm_ZoneEntropy | 0.058 |
| Narrowness | -0.036 |
| Headache | 0.069 |
| Hyperlipidemia | -0.027 |
| Hyperuricemia | -0.015 |
| Smoking | -0.092 |

*P < 0.05

Table 3. Class-specific performance metrics of the LR model on the independent test set

| **Class** | **No.of cases** | **Precision** | **Recall** | **F1-score** |
| --- | --- | --- | --- | --- |
| Recanalization | 28 | 0.926 | 0.893 | 0.909 |
| Non-recanalization | 5 | 0.500 | 0.600 | 0.545 |
| Weighted average | 33 | 0.861 | 0.848 | 0.854 |
|  |  |  |  |  |
